# Corticobasal syndrome: A diagnostic conundrum

**DOI:** 10.1590/s1980-5764-2016dn1004003

**Published:** 2016

**Authors:** Jacy Bezerra Parmera, Roberta Dieh Rodriguez, Adalberto Studart Neto, Ricardo Nitrini, Sonia Maria Dozzi Brucki

**Affiliations:** 1 Behavioral and Cognitive Neurology Unit, Department of Neurology, University of São Paulo, Brazil.

**Keywords:** corticobasal syndrome, corticobasal degeneration, dementia, atypical parkinsonism

## Abstract

Corticobasal syndrome (CBS) is an atypical parkinsonian syndrome of great interest to movement disorder specialists and behavioral neurologists. Although originally considered a primary motor disorder, it is now also recognized as a cognitive disorder, usually presenting cognitive deficits before the onset of motor symptoms. The term CBS denotes the clinical phenotype and is associated with a heterogeneous spectrum of pathologies. Given that disease-modifying agents are targeting the pathologic process, new diagnostic methods and biomarkers are being developed to predict the underlying pathology. The heterogeneity of this syndrome in terms of clinical, radiological, neuropsychological and pathological aspects poses the main challenge for evaluation.

## Abstract (pt)

A síndrome corticobasal é classificada dentro do grupo das síndromes parkinsonianas atípicas, e atualmente desperta interesse em neurologistas especialistas em distúrbios do movimento e neurologia cognitiva e comportamental. Inicialmente considerada como uma síndrome tipicamente motora, hoje se reconhece a importância dos achados cognitivos na apresentação, podendo ocorrer mesmo na ausência de alterações motoras. Tal designação refere-se à síndrome clínica e é associada a várias patologias subjacentes. Tendo em vista que drogas modificadoras da doença estão focando na patologia de base, novos métodos diagnósticos de imagem e outros biomarcadores estão sendo desenvolvidos para predizer o processo patológico específico antemortem. A heterogeneidade clínica e patológica desta entidade, portanto, é o maior desafio a ser desvendado.

## INTRODUCTION

In the vast group of neurodegenerative diseases, Corticobasal Syndrome was described particularly recently, in 1967 and 1968, when Rebeiz et al.^1,2^ first reported clinical and neuropathological features of three patients with a syndrome that they called "corticodentatonigral degeneration with neuronal achromasia". Gibb and Marsden used the term Corticobasal degeneration (CBD) in 1989^3^ and the term Corticobasal Ganglionic Degeneration has also been adopted by some authors. The clinical entity described by Rebeiz et al. is now considered an atypical parkinsonian syndrome of great interest to movement disorder specialists and behavioral neurologists and is referred to as Corticobasal Syndrome (CBS), denoting the clinical phenotype, and is associated with a heterogeneous spectrum of pathologies. The heterogeneity of this syndrome from clinical, radiological, neuropsychological and pathological aspects poses the main challenge for evaluation.

On the other hand, the pathologic entity CBD causes prominent focal cortical atrophy and subcortical damage and can be characterized with distinct clinical syndromes to CBS, such as Progressive Supranuclear Palsy (PSP), Frontal Behavioral-Spatial Syndrome (FBS), nonfluent/agrammatic variant of Primary Progressive Aphasia (naPPA), also presenting with a wide range of neurologic signs and symptoms. This review is based on a PubMed literature search from 1967 to date, and aims to provide an overview of the current knowledge on the corticobasal syndrome, covering six aspects: clinical features, biomarkers, imaging, pathology, genetics and treatment.

## CLINICAL FEATURES

The Corticobasal Syndrome is usually characterized by akinetic-rigid parkinsonism, dystonic and myoclonic movements, associated with cortical symptoms such as ideomotor apraxia, alien limb phenomena, aphasia or sensory neglect. There are many available criteria for CBS, and they differ considerably (Table 1).^4,5^ In the latest criteria, probable CBS is characterized by an asymmetric presentation with at least two of:

[a] limb rigidity or akinesia,
[b] limb dystonia,
[c] limb myoclonus, plus two of:
[d] orobuccal or limb apraxia,
[e] cortical sensory deficits,
[f] alien limb phenomena.^4^

In addition, other different cognitive deficits may coexist. Another study proposed a modified Cambridge criteria after comparing three previous criteria applied to a large group of 40 patients with a clinical diagnosis of CBS. As cognitive impairment was ubiquitous even at presentation, with speech and language impairment the commonest feature, the authors noted that all three criteria could be applied equally well at later stages, but in the earlier stages the Cambridge criteria had significantly wider applicability, almost certainly due to the weight given to cognitive and language dysfunction. Therefore, they suggested a minor modification to capture the high prevalence of aphasia (Table 1).^5^

**Table 1:** Current clinical criteria for CBS.

Modified Bak and Hodges criteria (Cambridge criteria) Mathew et al.^16^	Armstrong et al.^4^	
**Mandatory criteria** * • Insidious onset and gradual progression • No sustained response to levodopa treatment	**Probable** • Insidious onset/gradual progression • Asymmetric presentation	**Possible** • Insidious onset/gradual progression • May be symmetric
⭣	⭣	⭣
**Major criteria**	**Cortical dysfunction**	**Cortical dysfunction**
Motor features • *Akinetic rigid syndrome* Cortical motor sensory features • *Limb apraxia* Cognitive features • *Speech and language impairment *	At least 2 of: • Orobuccal/limb apraxia • Cortical sensory deficit • Alien limb phenomena	At least 1 of: • Orobuccal/limb apraxia • Cortical sensory deficit • Alien limb phenomena
**Minor criteria**	**Extrapyramidal dysfunction**	**Extrapyramidal dysfunction**
Motor features • Focal or segmental myoclonus • Asymmetrical dystonia Cortical motor sensory features • Alien limb phenomenon • Cortical sensory loss or dyscalculia Cognitive features • Frontal executive dysfunction • Visuospatial deficits	At least 2 of: • Limb rigidity or akinesia • Limb dystonia • Limb myoclonia	At least 1 of: • Limb rigidity or akinesia • Limb dystonia • Limb myoclonia

* For a diagnosis of CBS, the patient should satisfy all mandatory criteria, two major criteria (in italics) and two minor criteria.

With regard to motor presentation, including dystonia, rigidity, akinesia, myoclonus, tremor and levodopa-resistant parkinsonism, there is notable asymmetry. It is now recognized, however, that these motor features do not distinguish CBS underlying pathologies.^7-9^ In a recent study, involving 296 pathologically-proven cases of Corticobasal Degeneration, only 37.5% had dystonia, where upper limb dystonia was the most common pattern (77.4%), followed by cervical dystonia (9.5%) and blepharospasm (8.3%). CBS was present in 202 patients (54%), and of these cases, 51% had myoclonus, 86.3% apraxia and 100% had an akinetic-rigid syndrome. Considering this, despite dystonia being included in clinical criteria for CBS and CBD, this aspect does not seem to predict a clinicopathological correlation.^9^ Another study sought to investigate the frequency and pattern of dystonia in a group of patients with atypical parkinsonism. The series demonstrated dystonia as a common feature with overall frequency of 50%, and in the CBD group of 100% (8 patients). Dystonia was not the first complaint in any of these patients. Levodopa therapy did not influence the pattern of dystonia.^10^

Most studies describe myoclonus in the presentation of patients with CBS, occurring in 55% to 93% of cases,^11-13^ where terms used are "focal myoclonus" or "stimulus-sensitive myoclonus". This occurs commonly in upper extremities and can also be present in the face. They are typically spontaneous or triggered by sensory stimulation, and usually considered of cortical origin. Limb rigidity is commonly asymmetric and described as severe, but the nature is uncertain, and could be related to parkinsonism, dystonia or paratonia.^4^

Although commonly described as a "Parkinson-plus" syndrome, it is clear that behavioral and cognitive changes prevail in the clinical course, which may affect quality of life as much as the movement disorders. Initially considered an entity with primary damage to the basal ganglia and the frontal-parietal cortex, with parkinsonism and apraxia, recent investigations have shown variable involvement of frontal, parietal and temporal cortices, resulting in combinations of parkinsonism and other cognitive impairments. Higher cortical features include apraxia, alien limb phenomena, cortical sensory loss, global cognitive impairment, behavioral changes and aphasia.

Previously, cognitive deficits were considered a late-stage phenomenon.^14^ It is now known that these features are present from the outset of the illness, even in cases secondary to underlying CBD pathology, leading to their incorporation into most diagnostic criteria (Table 1). Occasionally, motor features emerge and patients later develop cognitive and language disturbances. The opposite is also observed; some patients with dementia syndrome criteria such as probable primary progressive aphasia (PPA), behavioral-variant frontotemporal dementia or posterior cortical atrophy, may develop motor features of CBS later in the course of the disease.

There is a bias in the frequency of reports on cognitive deficits, probable because these cases are commonly not evaluated by behavioral specialists, even though multidomain cognitive impairment is extensively reported in CBS patients. Patients can sometimes start the presentation with impairment in executive function and memory,^15^ but these symptoms are also commonly seen in other neurodegenerative diseases.

Deficits in language and visuospatial dysfunction seem to be much more characteristic. In some cases, language dysfunction is the first symptom, and most studies have reported reduced word fluency, speech apraxia and also syntactic deficits, when the CBS overlaps with the naPPA phenotype.^16,17^ A recent study using ligand Pittsburgh compound B-Positron Emission tomography (PiB- PET) imaging demonstrated a tendency for greater impairment of sentence repetition, also observed in logopenic progressive aphasia in PiB-Positive cases (PiB-positive 75%, PiB-negative 22.2%). Thus, the study suggested that impaired sentence repetition in CBS cases could predict AD pathology.^18,19^

Another large cohort study of 45 CBS patients demonstrated a frequent start with language impairment (69% of patients) compared to apraxia (29%), unlike most studies which highlight apraxia as the major cognitive sign of CBS. The predominant language impairment was coherent with asymmetrical hypoperfusion of left frontal-parietal and posterior temporal cortices. However, it is unclear whether there is a specific aphasia phenotype. There are findings of a phenotype suggestive of a "mixed" progressive aphasia, presenting with agrammatism and speech disorders, as well as with anomia and sentence repetition impairment (such as the logopenic variant of PPA) and disorders of single word comprehension (such as the semantic variant).^28^

Visuospatial dysfunction is also an alteration included in most diagnostic criteria for CBS.^7,17,18^ Some patients, who later go on to develop CBS, present Posterior Cortical Atrophy in the initial evaluation, and these deficits can be quite severe. They can develop Balint's Syndrome or only one of the components of the syndrome (simultanagnosia, oculomotor apraxia and optic ataxia),^22^ and can also develop Gerstmann's syndrome (dyscalculia, dysgraphia, finger agnosia and left-right disorientation) or visual agnosia. A cohort study demonstrated the existence of Gerstmann's syndrome as a frequent finding in CBS cases related to a probable AD underlying cerebrospinal fluid (CSF) signature, with considerable sensitivity (75%) and specificity (75%).^28^

The assessment of visuospatial functions in CBS and atypical parkinsonism syndromes has to overcome a wide range of confounding variables.^23^ The exact frequency of visuospatial deficits and the interpretation of existing studies are complicated by the possible influence of motor and frontal executive deficits, and are therefore difficult to measure, because these motor deficits and other higher cortical dysfunction sometimes can render the examination almost impossible. A test called the Visual Object and Space Perception Battery (VOSP) was used in one study to minimize the influence of motor and executive dysfunction and to distinguish between object and space processing alterations (ventral and dorsal streams, respectively). The percentage of patients impaired ranged from 28% to 52%, with spatial tests more often impaired (44-52%) than object-based tests (28-38%), suggesting early involvement of the "dorsal stream" with its anatomical substrate in parietal lobe pathology.^24^ Another study using PiB binding has shown a correlation of performance on the VOSP in CBS with underlying Alzheimer's pathology.^19^

There are two disorders of voluntary action included in diagnostic criteria and normally present in clinical features of CBS, namely, alien limb and apraxia. Both archetypical disorders of volition, the first represents a performance of semi-purposeful movements in the absence of volition.^24^ The phenomenon of alien hand syndrome is complex and has various clinical manifestations related to different lesion sites, such as supplementary motor area, anterior cingulate, corpus callosum, anterior prefrontal cortex, posterior parietal cortex and thalamus.^26^

Case studies have associated lesions in the anterior corpus callosum with volitional disorders of alien limb and apraxia in the non-dominant hand. Damage to this tract could lead to compromised transition of sensorimotor signals from the dominant to the non-dominant hemisphere. Thus, apraxia and alien limb could represent a "disconnection syndrome", as sensorimotor representations for voluntary movements are disconnected from motor areas.^25^ There is evidence from studies regarding volitional deficits of alien limb and apraxia, considering that they both can occur in the same patient but are dissociable, and correlating focal structural changes in gray and white matter of the medial frontal-prefrontal network and its connectivity with the pre-supplementary motor area.^22^ Another study utilizing functional magnetic resonance imaging showed an association of alien limb and a broader network of brain regions related to movement execution and planning as well as areas linked to inhibition control, the inferior frontal gyrus and the precuneus. Behavioral symptoms similar to those observed in patients with behavioral variant^26^ frontotemporal dementia may be present, typically apathy rather than disinhibition.^18^

Akin to motor features, there are no cognitive or language manifestations that reliably distinguish between underlying pathologies in patients with CBS.^11^

Other symptoms not usually related to CBS but likely important to quality of life have also been studied. Swallowing and speech disturbances are common in these patients and differ from the same symptoms in other parkinsonian syndromes such as PSP. Speech apraxia and piecemeal deglutition is also a characteristic feature in CBS.^27^

## PATHOLOGY

The constellation of CBS is associated with a variety of underlying pathologies other than CBD. Many patients with post-mortem diagnosis of CBD are never suspected of having the disease during life.^1,6,8,35^ Additionally, CBD pathology was found in only 50% of all clinically diagnosed patients, with others showing PSP, Pick's disease, FTLD- TDP43, AD, dementia with Lewy bodies, and Creutzfeldt-Jakob disease at autopsy.^2,6,8,35-37^ Due to this clinical-pathologic diversity, Boeve et al. (2003) introduced the term CBS to distinguish the clinical syndrome from the pathologic entity, CBD.^38^

Diagnosis of the underlying cause of CBS is only possible through postmortem brain analysis due to the degree of clinical-pathologic mismatch that exists. The majority of causes of CBS are tauopathies.^39^ In the 1990s, the neuronal aggregates in CBD^40^ were shown to consist of the microtubule associated protein (MAPT). The tau protein exists in 6 isoforms as a result of alternative mRNA splicing of the exons 2, 3 and 10. The inclusion of exon 10 generates an isoform with four microtubule-binding domains (4R), while the absence of this inclusion produces an isoform with three microtubule-binding domains (3R). The different neurodegenerative disorders that can cause CBS have been associated with specific tau isoforms. CBD features predominant deposition of 4R-tau, and likewise PSP, while AD is characterized by the simultaneous presence of 3R and 4R-tau protein, and Pick's disease by 3R-tau.^41^

**Figure 1:**
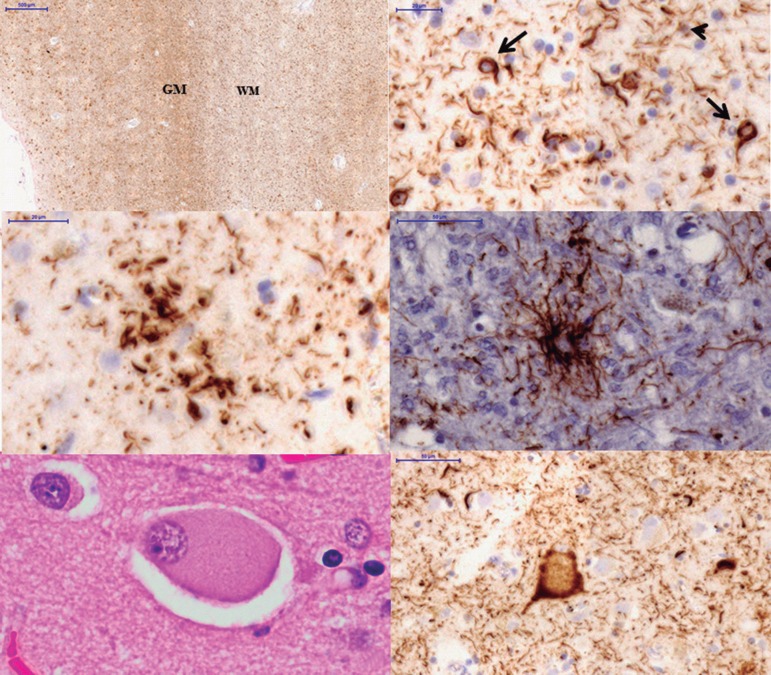
Microscopic findings of CBD and PSP: [A] boundary between GM and WM in the inferior temporal gyrus of a CBD case. Note the severe involvement of both compartments (tau immunostain, CP13 antibody); [B] oligodendroglial coiled bodies (arrows) and thread-like pathology (arrowhead) in white matter in CBD case (tau immunostain, CP13 antibody); [C] astrocytic plaque, a hallmark of CBD (tau immunostain, CP13 antibody); [D] tufted-astrocyte, the characteristic glial lesion of PSP (tau immunostain, CP13 antibody); [E] ballooned neuron in temporal cortex (hematoxylin eosin); [F] tau-positive ballooned neuron in temporal cortex. Scale bars represent 500 µm in A; 20 µm in B, C; 50 µm in [D, E]; and 10 µm in [F]. GM: gray matter; WM: white matter.

Besides these distinct biochemical features, microscopically some findings could help to distinguish the different pathologic causes of CBS. Neuropathological diagnostic criteria for CBD require tau inclusions in neurons and glia with astrocytic plaques and extensive thread-like pathology.^42^ Like CBD, PSP has threads in gray and white matter, but in CBD the boundary between gray and white matter may be indistinct due to the severity of threads in both compartments (Figure 1).^39,44,45^

Astrocytic plaques are the hallmark glial lesion of CBD and the most distinguishing histopathological feature of CBD and PSP. Astrocytic plaques represent tau accumulation in the distal segments of astrocytes with minimal accumulation in the cell body, creating a central clear zone (Figure 1). They are more numerous in cortex, but can also be seen in caudate and putamen and less often in thalamus and midbrain tectum.^39,43-45^ By contrast, in PSP the characteristic glial lesion is the tufted astrocyte (Figure 1). They are seen especially in the precentral gyrus, striatum and superior colliculus, being more variable in the thalamus, subthalamic nucleus and red nucleus yet rare or absent in the lower brainstem. A third neuropathological lesion highly suggestive of CBD is the ballooned neuron (BN) (Figure 1). These are swollen cortical neurons, most often found in the third, fifth and sixth cortical layers, and have been linked to chromatolysis. Cingulate gyrus, amygdala, insular cortex and claustrum are the most common locations.^39,44,45^ Unlike in argyrophilic grain disease, limbic and paralimbic distribution of BN should not be considered specific of CBD. In addition, the presence of BN in convexity cortical areas is of much more diagnostic significance. BN are rare or absent in PSP.^39,44,45,47^

In addition, the presence of oligodendroglial tau inclusions called coiled bodies are common in CBD, but are much more frequent in PSP than CBD (Figure 1). In PSP they tend to be parallel to the distribution of neuropil threads and can be numerous in white matter tracts in the basal ganglia, thalamus and brainstem.^39,44,45,47^

Predicting underlying pathology in CBS is difficult, because of the multitude of etiologies. The sensitivity of clinical findings for predicting underlying CBD pathology ranges from 26% to 56 %.^45-47^ Lee et al. (2011) observed a 35% prevalence of CBD post-mortem in 40 patients meeting CBS criteria, followed by 23% AD, 13% PSP and 13% FTLD-TDP43.^8^

Another study of Ling et al. (2010) involving a movement disorder-focused series, found a frequency of 53% CBD or PSP, and 24% AD in 21 cases clinically diagnosed with CBS. The clinical presentation and progression of symptoms reflect the distribution of the pathology more than the specific underlying histology (Table 2).^35^

**Table 2:** CBS: Pathologic correlations.

Study Pathology	Boeve, 2003^37^	Hodges, 2004^58^	Josephs, 2006^59^	McMonagle, 2006^29^	Shelley, 2009^6^	Ling, 2010^34^	Lee, 201^17^	Total, n (%)
CBS cases, n	34	9	21	19	12	21	40	156 (100)
CBD	18	7	10	11	6	5	14	71 (45.5)
PSP	6	0	10	1	0	6	5	28 (18.0)
AD	3	0	0	1	6	5	9	24 (15.4)
Pick’s disease	2	0	1	3	0	0	1	7 (4.5)
DLDH	2	1	0	0	0	1	0	4 (2.6)
PD	0	0	0	0	0	2	0	2 (1.3)
FTLD-TDP43	0	1	0	2	0	1	5	9 (5.8)
FTLD-TDP43 + MND	0	0	0	0	0	1	0	1 (0.6)
CJD	3	0	0	1	0	0	0	4 (2.6)
MST	0	0	0	0	0		1	1 (0.6)
Mixed disease^a^	0	0	0	0	0	0	5	5 (3.2)

a Mixed cases: 2 PSP, 1 CBD, 1 FTLD-TDP, all mixed with intermediate probability of Alzheimer’s disease. CBD: Corticobasal degeneration; PSP: Progressive supranuclear palsy; AD: Alzheimer’s disease; DLDH: Dementia lacking specific histology; PD: Parkinson’s disease; FTLD-TDP43: Frontotemporal lobar degeneration with TDP-43 inclusions; MND: Motor neuron disease; CJD: Creutzfeldt- Jacob disease; MST: Multiple system tauopathy without argyrophilia.

The better characterization of clinical, neuropsychological and imaging features is important to improve antemortem diagnosis and crucial for designing therapies.

## IMAGING AND BIOMARKERS

There is growing interest in developing disease-specific biomarkers to aid the prediction of pathology in the antemortem diagnosis of neurodegenerative disorders. Tau and Alzheimer disease pathology-targeted therapies are currently being developed and undergoing clinical trials. Therefore, determining the nature of the underlying pathology ought now to be considered of great importance and not only a matter of purely academic interest. The possible future biomarkers include CSF testing and imaging modalities.^11^ Although the pathophysiology of CBS is largely unknown, recent advances in neuroimaging have shed light on specific structural neuroanatomical changes that occur as a result of this disorder.^31^

A recent study assessed gray matter and white matter changes using a more advanced technique than voxel-based morphometry (VBM), the surface-based morphometry, which evaluates cortical thickness and surface area, by also using diffusion tensor imaging (DTI) to evaluate white matter. The results showed that cortical thinning, subcortical volume loss and fiber tract degeneration prominently involved the hemisphere contralateral to the more affected limb. These findings corroborate other data suggesting that the asymmetric distribution affecting frontostriatal connectivity is closely associated with asymmetric motor and non-motor symptoms.^31^ The patterns of white matter damage in bvFTD and CBS have been contrasted using DTI. They showed greater damage to the uncinated fasciculus, genu of corpus callosum and forceps minor. In contrast, CBS patients had greater damage to the midbody of the corpus callosum and perirolandic corona radiata, thus the distribution and degree of white matter damage differed between them.^32^

Apparently, specific patterns of atrophy may suggest underlying pathology. Volumetric Magnetic Resonance Imaging (MRI) using VBM was used to compared groups of CBS with different postmortem diagnosed pathologies, such as CBS-AD (6 cases), CBS-CBD (7 cases), CBS-PSP (6 cases) and CBS-FTLD-TDP43 (frontotemporal lobar degeneration with TDP-43 inclusions; 5 cases), revealing that imaging patterns of gray matter loss differ according to the underlying pathology. All CBS pathologic groups showed gray matter loss in premotor cortices, the supplemental motor area and insula on imaging. CBS-TDP43 and CBS-AD were associated with a more widespread pattern of gray matter loss, and in CBS-TDP43 cases there was predominantly loss in the prefrontal cortex and posterior temporal lobes. The CBS-AD group was associated with a posterior pattern of gray matter imaging loss, involving parietal, posterior temporal and occipital lobes.^33,34^

Also, a representative cohort population of 45 CBS patients was analyzed for AD profile in CSF with biomarkers, a possible useful tool to distinguish between underlying pathologies, together with brain perfusion imaging (Spect). It disclosed two distinct anatomo-clinical variants, both related (18% of cases) and unrelated (82%) to probable underlying AD. AD-CBS cases were more frequently characterized by myoclonus and Gerstmann syndrome, whereas non-AD CBS more frequently had orobuccal apraxia and severe aphasia. Spect imaging showed that AD-CBS involved posterior parietal-temporal cortices, pre-cuneus and posterior cingulate, while non-AD CBS had more anterior damage to left-sided frontal cortices impacting language. These findings raised the question as to whether some CBS should be considered atypical AD.^28^ Tau/Abeta ratio in CSF, as used in this study, may represent a useful way of detecting CBS-AD in vivo, although neuropathological confirmation is not yet forthcoming and the technique's sensitivity for detecting CBS-AD at an early stage has yet to be determined.^48^

Recently, the use of specific ligand-based nuclear imaging modalities such as PiB-PET, which was developed to detect fibrillary b-amyloid peptide and is a sensitive and specific biomarker of AD pathology, can be used to detect pathology in vivo in patients with dementia syndromes and can distinguish different neurodegenerative disorders.^18^ Amyloid imaging may be of value to determine which cases are related to AD, although it is known that 15%-30% of cognitively-normal older individuals can have a positive amyloid PET.^47^ Tau-ligand imaging is also the subject of current research and probably will be incorporated in the future to predict pathology. Reflecting the rarity of the disease, the number of participants in these studies of ligands to amyloid or tau typically remains small.

The first study to use amyloid imaging in CBS included 14 CBS patients to undergo PiB-PET imaging, four (28.6%) were PiB-positive –patients with high PiB binding, a standardized uptake ratio >1.5- and the remaining were PiB-negative (71.4%). There were no significant differences in motor examination findings between the two groups, though sentence repetition impairment revealed a tendency for greater impairment in PiB-positive cases, and also for visuospatial function, memory impairment and everyday skills domains. VBM analyses showed atrophy affecting the posterior part of the left superior temporal gyrus, distinguishing PiB-positive cases.^18^

Another more recent study using amyloid imaging split CBS into frontal and temporoparietal clinical variants based on modified clinical criteria, MRI and FDG-PET and compared with PiB-PET results. In total, 25 patients underwent amyloid imaging, and nine out of the fourteen patients classified as temporoparietal variant were PiB-positive (82% sensitivity and 71% specificity). Cognitive testing demonstrating greater episodic memory and visuospatial impairment than executive dysfunction had the strongest association with PiB status.^29^

Temporoparietal-predominant neuroimaging patterns with FDG-PET hypometabolism proved sensitive but not specific for AD. One autopsy-proven patient with a positive amyloid PET scan had the presence of CBD pathology, indicating that the possibility of co-pathology must be considered.^30^ Amyloid PET scans, although an optimal modality for detecting AD pathology in CBS patients, is not widely available and further knowledge about more accessible neuroimaging modalities is still required.

## GENETICS

The genetics of cases of CBS is largely unknown and the majority are sporadic. CBS, when genetically related, is frequently observed in patients who have mutations in the gene that encodes progranulin (PGRN).^50^ There have been reports of families with autosomal-dominant frontotemporal lobar degeneration linked to PGRN gene mutations that could represent 5-7.9% cases in large series of CBS.^52^

Mutation in the MAPT gene has also been demonstrated in a CBS-like presentation^52^ as well as pathogenic C9orf72 repeat expansion, particularly when there is a positive family history of FTD and amyotrophic lateral sclerosis (ALS).^53^

A recent case report described a family with pathologically-confirmed cases of early-onset, autosomal-dominant familial AD (EOFAD) linked to a Met233Leu mutation of the presenilin-1 gene (PSEN-1), and one family member developed prominent CBS combined with severe neuropsychiatric and behavioral disturbances resembling those often found in EOFAD. The authors concluded that CBS may represent an atypical clinical presentation in autosomal-dominant EOFAD and that the PSEN-1 gene could be an opportunity to predict AD pathology. They also suggested testing for PSEN-1, PGRN, MAPT and C9orf72 gene mutations when there is a positive family history of neurodegenerative conditions.^54^

Another study suggested testing genetic mutation in FTLD with movement disorders as a motor presentation, and when CBS is present, testing first for PGRN and after, if the first is negative, testing for MAPT, C9orf72, CHMP2B (which encodes charged multivesicular body protein 2b), VCP (valosin-containing protein), FUS (which encodes RNA-binding protein FUS- fused in sarcoma), TARDBP (TAR DNA-binding protein 43) and NIFID (neuronal intermediate filament inclusion disease).^50^

## TREATMENT

There is no specific treatment for CBS, but the ability to accurately detect underlying pathology early in the course of CBS will be crucial when effective therapies are developed.^18^

Symptomatic treatment of CBS is used to improve motor and cognitive-behavioral symptoms, but in general these are largely based on Class IV evidence, due to lack of randomized clinical trials.^55^ Levodopa can be helpful in CBS, as demonstrated in an observational study which showed that 56% of pathologically-confirmed CBD patients had slight improvement in bradykinesia and rigidity. To consider a subject as a non-responder, it is recommended to treat the individual with a dosage of 1000 mg daily for at least 2 months before withdrawal.^35,36^ Although no definitive data are available regarding the efficacy of botulinum toxin (BoNT) type A and B, it may be helpful for CBS-associated limb dystonia and may be used to alleviate abnormal posture, pain and for maintaining hand hygiene.^55,57^ Usual therapeutic strategies for myoclonus include levetiracetam (up to 3000 mg/day) or benzodiazepines (clonazepam, up to 15mg/day).^55^

With regard to cognitive and behavioral symptoms, acetylcholinesterase inhibitors can be considered for patients with CBS that may have underlying AD pathology. For psychosis, agitation and aggression, anti-psychotics (atypical agents) are employed despite adverse effects that include extrapyramidal symptoms. Mood stabilizers, such as carbamazepine and valproic acid, can be used to control agitation. Trazodone has been employed for behavioral symptoms in FTLD, but in CBS no clear data on its effectiveness are available, and selective serotonin reuptake inhibitors (SSRIs) provide effective treatment in these subjects.^55^ In a case report, alien hand syndrome was highly responsive to amantadine.^58^

Non-pharmacological therapies, such as cognitive behavioral therapy, physiotherapy, occupational therapy, are employed in CBS patients, improving quality of life, as well as motor, speech and language symptoms.

Disease-modifying agents targeting the pathologic process are undergoing development, highlighting the importance of accurate pathological diagnosis in the near future.

## CONCLUSION

CBS is an enigmatic diagnosis, as a syndrome with many motor and non-motor symptoms due to different underlying pathologies, still not accurately diagnosed in vivo. The wide range of cognitive, behavioral and motor aspects is extremely variable between patients.

Further characterization of the clinical, imaging and neuropsychological hallmarks of CBS patients related to specific pathology is very important, considering the new recent advances in treatment. Patients with underlying AD pathology and tauopathies correctly diagnosed in the future may benefit from symptomatic therapies and future disease-modifying agents.
